# Spatial constraints and cognitive fatigue affect motor imagery of walking in people with multiple sclerosis

**DOI:** 10.1038/s41598-020-79095-3

**Published:** 2020-12-14

**Authors:** Jessica Podda, Ludovico Pedullà, Margherita Monti Bragadin, Elisa Piccardo, Mario Alberto Battaglia, Giampaolo Brichetto, Marco Bove, Andrea Tacchino

**Affiliations:** 1 Scientific Research Area, Italian Multiple Sclerosis Foundation (FISM), Genoa, Italy; 2 AISM Rehabilitation Service, Italian Multiple Sclerosis Society, Genoa, Italy; 3 Department of Physiopathology, Experimental Medicine and Public Health, University of Siena, Siena, Italy; 4 Department of Experimental Medicine, Section of Human Physiology and Centro Polifunzionale di Scienze Motorie, University of Genoa, Genoa, Italy; 5 IRCCS Ospedale Policlinico San Martino, Genoa, Italy

**Keywords:** Psychology, Human behaviour, Multiple sclerosis, Sensorimotor processing

## Abstract

Motor imagery (MI) is the mental simulation of an action without any overt motor execution. Interestingly, a temporal coupling between durations of real and imagined movements, i.e., the so-called isochrony principle, has been demonstrated in healthy adults. On the contrary, anisochrony has frequently been reported in elderly subjects or those with neurological disease such as Parkinson disease or multiple sclerosis (MS). Here, we tested whether people with MS (PwMS) may have impaired MI when they imagined themselves walking on paths with different widths. When required to mentally simulate a walking movement along a constrained pathway, PwMS tended to overestimate mental movement duration with respect to actual movement duration. Interestingly, in line with previous evidence, cognitive fatigue was found to play a role in the MI of PwMS. These results suggest that investigating the relationship between cognitive fatigue and MI performances could be key to shedding new light on the motor representation of PwMS and providing critical insights into effective and tailored rehabilitative treatments.

## Introduction

Motor imagery (MI) is an active mental process in which a subject internally simulates a movement without any corresponding motor output. Accumulating evidence indicates functional similarities between actually and mentally executed actions, notably regarding temporal characteristics, neural correlates and autonomic responses^1^. A plethora of studies shows that actual and mental movements are subject to common motor rules and principles (e.g., Fitts’ law)^2–4^. Indeed, results on healthy adults usually indicate a temporal coupling between the duration of actual and mental movements^3, 5,6^, the so-called “isochrony principle”^2^. Congruently, fMRI studies have shown that the neural networks involved in MI overlap considerably with those recruited for actual motor execution^7–9^.

On the contrary, anisochrony, i.e., a temporal discrepancy between actual and mental movements, has been frequently reported in populations of neurological patients, as people after stroke^10^, with Parkinson disease (PD)^11,12^ and with multiple sclerosis (MS)^13–15^, gaining increasing attention as a promising additional clinical investigation tool^16^. Recently, Tacchino et al.^17^ showed that behavioural performance and brain activations during MI are correlated to disease severity in people with MS (PwMS) and proposed anisochrony as a surrogate behavioural marker of MS evolution, especially in the early stages of the disease.

As for healthy subjects (HS), most investigations on MI of people with neurological diseases have mainly examined upper limb movements with different motor tasks such as sequential pressing of buttons^18^, finger-to-thumb opposition^19^, pointing towards targets^15,20^, hand manipulation^17,21^, hand grasping and arm-lifting^22^, and bimanual circle-line coordination^23^. However, examples of MI involving lower limb are also present^24–30^.

Walking is particularly interesting for a broader understanding of MI. In fact, this represents a unique window into the study of MI because subjects are requested to simulate full-body movements and simultaneously update environmental spatial information^5^. In general, findings indicate that healthy good imagers usually preserve temporal congruence^31–33^ as well as recruiting very similar cerebral networks (e.g. fronto-parietal areas, basal ganglia, brainstem, cerebellum)^34,35^ when they are asked to actually and mentally execute tasks involving locomotion.

Since impaired locomotion is a frequent and major source of disability in patients with neurological diseases and new techniques are continuously required to improve gait rehabilitation, a better characterization of MI of locomotion in these populations is fundamental. People after stroke seem to retain locomotion MI abilities^36^ and mental practice appears to be a beneficial intervention for stroke rehabilitation^37^; nevertheless the existing evidence remains inconclusive because of significantly statistical heterogeneity and methodological flaws^38^. More contradictory results have been found in patients with PD, mainly due to differences in testing procedures and task instructions^27,39,40^.

The few studies investigating MI in PwMS are mainly limited to considering upper limb actions^13,15,17^, showing also how cognitive and mood disorders affect MI^28,41,42^. Only recently the focus has been shifted to lower limb movements^43–45^. Interestingly, evidence indicates that MI has been shown to improve not only walking, but also balance, fatigue, mood and quality of life in MS^43–45^. However, to date, it remains unclear whether PwMS show anisochrony between actual and mental locomotion as well and, if so, whether this temporal discrepancy is modulated by spatial constraints^5,13,15,17^ and/or affected by cognitive fatigue.

Investigating the relationship between fatigue and MI performances in PwMS could be key to shedding new light on the motor representation of PwMS and providing insights into new rehabilitative treatments. In fact, fatigue is one of the most debilitating symptoms in MS as it significantly impacts patients’ daily life activities and quality of life^46^. Given its multifactorial nature, frequently divided into various components such as physical vs. cognitive, fatigue is difficult to define or operationalize, although neurophysiological and fMRI studies have indicated that a cortico-subcortical disconnection could support the central origin of fatigue and explain its complex interaction with other clinical conditions (e.g. mood, cognitive functioning, disability)^47^. Interestingly, given the significant cognitive effort required to concentrate on MI tasks, loss of attention and declined arousal level due to cognitive fatigue have been found to significantly alter neural signals in the Brain Computer Interface (BCI) system^48^. As indicated by Deluca et al.^49^, abnormal cerebral activation in the basal ganglia and frontal lobes (that are also recruited in the MI task) may represent the extra “effort” (i.e., allocation of more neural resources) required to maintain the same level of performance, supporting the notion that increased cerebral activation may reflect the additional effort (i.e., cognitive fatigue) to adequately perform behavioral tasks in PwMS. Therefore, investigation of the complex inter-relationship between cognitive fatigue and MI is very relevant.

Although there is clear and consistent evidence that PwMS may have difficulties in the mental simulation of actions and that several aspects could affect performances, previous results are far from conclusive. Thus, to shed light on this aspect, we asked PwMS to actually and mentally walk along three paths with different widths and we compared their performances to those of HS.

## Methods

### Participants

Sample size was determined using values from the study by Tacchino et al.^17^ which suggested a 0.3 point difference [standard deviation (SD) = 0.3] in Index of Performance (IP), a measure of participants’ mental movement ability, of PwMS. Considering 80% power and 5% (two-sided) level of significance, the planned sample size was 15 PwMS and 15 HS. PwMS were recruited among outpatients at the Rehabilitation Service of Genoa of the Italian Multiple Sclerosis Society (AISM). Inclusion criteria for PwMS were clinically defined MS according to McDonald criteria^50^, a stable phase of the disease without relapses in the previous 3 months, all disease courses, an Expanded Disability Status Scale (EDSS) ≤ 6.5 (i.e. able to walk with at most one aid, e.g. cane or single crutch)^51^. The HS were age-matched, did not present any neurological or muscular disorders and had normal or corrected-to-normal vision. Moreover, the subjects belonging to the two groups were included if they were right-handed as determined by the Edinburgh Handedness Inventory^52^ and showed preserved cognitive functioning as measured by a Symbol Digit Modalities Test^53^ (SDMT) score > 38. Subjects with a history of severe psychiatric disorders as indicated by Diagnostic and Statistical Manual of Mental Disorders, Fifth Edition (DSM-5) criteria^54^, blurred vision or cardiovascular and respiratory disorders were excluded. Participants from both groups were assessed with the following tests: the Timed 25-Foot Walk (T25-FW)^55^ to measure performances in walking speed, the short form of the International Physical Activity Questionnaire (IPAQ)^56,57^ to gather information about the usual time spent in walking, in vigorous and moderate intensity activity, and in sedentary behaviours, the Dual-tasking questionnaire (DTQ)^59^ which collects information about everyday difficulties with dual-tasking and the Kinesthetic and Visual Imagery Questionnaire (KVIQ)^58^ for MI vividness. PwMS were also assessed with the Modified Fatigue Impact Scale (MFIS)^60^ to gather information about fatigue perception.

All subjects gave written informed consent in accordance with the revised Declaration of Helsinki^61^. The study was approved by the Ethics Committee of San Martino Hospital, Genoa, Italy. All methods were carried out in accordance with relevant guidelines and regulations.

### Procedure

**Figure 1:**
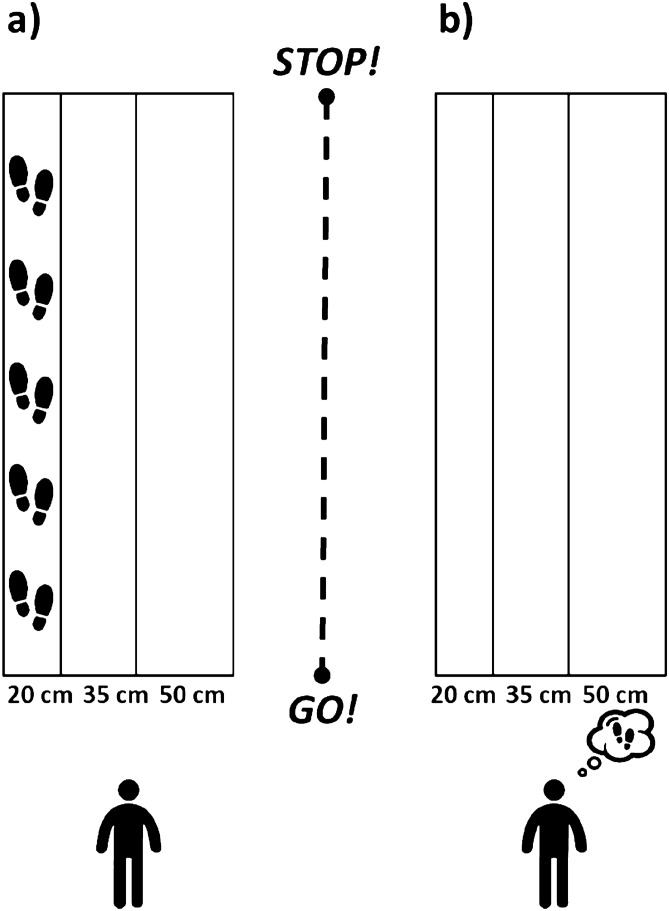
Experimental setup. Both groups of participants were asked to actually (**a**) or mentally (**b**) walk through paths of 5 m of length and delimited at three different widths: 20 cm, 35 cm, 50 cm. Icons were made by Freepik from www.flaticon.com.

The experimental setup was an adapted version from Personnier et al.^5^. Participants stood upright behind the starting line; their arms were hanging along the body, and their feet were parallel and slightly apart. They were asked to actually or mentally walk through three 5-m long paths, each with a different width: 20 cm, 35 cm and 50 cm. The different spatial constraints made it possible to test walking under several conditions, from normal to very accurate according to the path’s width. During actual movements, participants were invited to perform the task without walking on the lines limiting the paths at the self-paced speed. During mental movements, participants had to imagine themselves walking at a self-paced speed in the first-person perspective (i.e., as if one were the actor of the action). This has been considered to be closer to the real execution of movement^14^ since individuals elicit kinaesthetic sensation representations of actions as if they were actually performing them (see Fig. 1 for a representation of the experimental setup).

The duration of actual and mental movements was recorded by means of an electronic stopwatch (temporal resolution: 10 ms). When the experimenter gave the “Go!” signal, temporal acquisition started, and participants had to begin walking or imagining they were walking. During the actual movements, the temporal acquisition was stopped when the participants’ shoulders clearly crossed the finish line; during mental trials participants were required to verbally indicate “Stop!” when they mentally crossed the finish line. The time elapsed between the “Go!” signal and crossing the line corresponded to the movement duration.

Participants performed 12 walking movements for each path width (20 cm, 35 cm and 50 cm) in each experimental condition (actual and mental); for a total of 72 movements distributed over two different sessions separated by a time interval of 48 h. Within each session, actual and mental trials were presented to the participants in two different blocks. In the first session, the mental movements were performed before the actual ones, while in the second session the actual movements were performed before the mental ones. The three paths were randomly presented to the participants within each block. The two experimental conditions were separated by a 5-min break. In order to familiarize with the task, all participants performed one actual and one mental movement within each path. No information concerning their temporal performance was given to the participants during the practice or the experimental trials.

### Statistical analysis

For each actually (A) and mentally (M) executed path, the mean movement duration of locomotion was calculated. In order to better examine participants’ mental movement ability, we computed an Index of Performance (IP) expressed in percentage:

$$IP=\frac{A-M}{A} x 100$$

The IP reflects whether participants overestimate (negative values) or underestimate (positive values) movement durations during MI with respect to actual performances, where an IP approaching zero would represent a good motor imagery performance. However, although it is considered a reliable and objective measure of MI accuracy and an indicator of motor system integrity^7,62^, this index could hinder differences between imagined and actual movement durations when, for instance, some subjects underestimate and others overestimate movement durations during MI. As suggested by^5^, in order to provide a clearer and more complete analysis of MI performance, we also calculated the absolute values of IP.

A Shapiro–Wilks test revealed that all variables were normally distributed (always *p* > 0.05) and, therefore, parametric tests were used. Independent t tests were performed on SDMT, T25FW, IPAQ, DTQ and KVIQ total scores to assess potential differences between groups in cognitive, locomotion, usual physical abilities, dual-task and MI vividness performance. A two-way analysis of variance (ANOVA), with *group* as a between-subjects factor (PwMS; HS) and *path width* as a within-subjects factor (20 cm; 35 cm; 50 cm) was performed on IP values. Furthermore, an ANOVA with *group* as a between-subjects factor (PwMS; HS), *path width* (20 cm; 35 cm; 50 cm) and *type of movement* (actual; mental) as within-subjects factors was performed on actual and mental movement durations. Post-hoc tests (Bonferroni’s correction; *p* < 0.05) were applied to explore significant effects and interactions. To test any potential association between anisochrony and fatigue perception among PwMS, a correlation analysis was performed between the absolute values of IP (IP_abs_)^5^ across different path widths and MFIS (total score and subscale scores). Statistical analyses were run using SPSS 23.

## Results

In order to evaluate MI in PwMS, we asked fifteen PwMS (14 with relapsing–remitting course; 1 with secondary progressive course; 4 males; mean EDSS: 2.97 ± 1.86; mean MFIS: 38.53 ± 17.57) and fifteen HS (7 males) to take part in this study. The two groups were age matched (PwMS: 42.67 ± 11.67 years; HS: 41.60 ± 13.35 years; t28 = 0.23; *p* = 0.82). No significant differences were found between groups in walking speed as indicated by the T25-FW (PwMS: 7.14 ± 1.93 s; HS: 6.23 ± 1.03 s; t28 = 1.59; *p* = 0.122), in usual physical activity by IPAQ (walking in MET-min/week: PwMS: 1178.10 ± 519.19; HS: 1214.40 ± 208.78; t28 = -0.25; *p* = 0.803; moderate in MET-min/week: PwMS: 1420 ± 949.13; HS: 1536 ± 586.48; t28 = − 0.40; *p* = 0.690; vigorous in MET-min/week: PwMS: 1760 ± 1146.22; HS: 1872 ± 422.56; t28 = − 0.35; *p* = 0.725; total in MET-min/week: PwMS: 4358.10 ± 2366.04; HS: 4622.40 ± 820.83; t28 = − 0.41; *p* = 0.686; sedentary in h/day: PwMS: 4.4 ± 2.2; HS: 3.4 ± 1.40; t28 = 1.48; *p* = 0.149), in dual-task abilities as evaluated by DTQ (PwMS: 0.16 ± 0.07; HS 0.13 ± 0.07; t28 = 1.00; *p* = 0.326), in information processing speed measured by SDMT (PwMS: 48.27 ± 19.34; HS: 51.00 ± 11.56; t28 = − 0.47; *p* = 0.64) and in MI vividness as tested by KVIQ (PwMS: 126.20 ± 33.28; HS: 137.53 ± 21.47; t28 = − 1.11; *p* = 0.28). See Table 1 for a summary.

**Table 1:** Participants’ demographic and clinical characteristics.

	PwMS (n = 15)	HS (n = 15)	p values	Cohen's d
Age (y)	42.67 ± 11.67	41.60 ± 13.35	0.817	0.08
EDSS	2.97 ± 1.86			
MFIS	37.87 ± 15.56			
SDMT	48.27 ± 19.34	51.00 ± 11.56	0.642	0.17
T25-FW (s)	7.14 ± 1.93	6.23 ± 1.03	0.122	0.59
IPAQ				
Walking (MET-min/week)	1178.10 ± 519.19	1214.40 ± 208.78	0.803	0.09
Moderate (MET-min/week)	1420 ± 949.13	1536 ± 586.48	0.690	0.15
Vigorous (MET-min/week)	1760 ± 1146.22	1872 ± 422.56	0.725	0.13
Total (MET-min/week)	4358.10 ± 2366.04	4622.40 ± 820.83	0.686	0.1
Sedentary (h/day)	4.4 ± .2.2	3.4 ± 1.4	0.149	0.54
DTQ	0.16 ± .07	0.13 ± .07	0.326	0.36
KVIQ	126.20 ± 33.28	137.53 ± 21.47	0.277	0.41

Mean values and standard deviations are shown.

*HS* healthy subjects, *PwMS* people with multiple sclerosis, *EDSS* Expanded Disability Status Scale, *MFIS* Modified Fatigue Impact Scale, *SDMT* Symbol Digit Modalities Test, *T25FW* Timed 25-Foot Walk, *IPAQ* International Physical Activity Questionnaire, *DTQ* Dual-Tasking Questionnaire, *KVIQ* Kinesthetic and Visual Imagery Questionnaire.

### Overestimation of mental movement duration in PwMS

The results of an ANOVA on IP showed a main effect of *group* [F (1,28) = 12.79, *p* = 0.001, η2 = 0.314]. IP approached zero at all path widths in HS (− 3.5% ± 4.7%), while PwMS tended to mentally overestimate the duration of the required action (− 27.3% ± 4.7%), reflecting the presence of anisochrony (i.e. mentally executed locomotion lasted longer than actually executed locomotion). No significant differences in the *path width* factor (*p* = 0.456, η2 = 0.028) or in group*path width interaction (*p* = 0.103, η2 = 0.078) were found. However, we observed a trend towards anisochrony in PwMS with the decreasing of the path width (PwMS M ± SE: 20 cm: − 29.6% ± 4.6%, 35 cm: − 27.9% ± 5.1%, 50 cm: − 24.3% ± 5.0%; HS M ± SE: 20 cm: − 1.9% ± 4.6%, 35 cm: − 4.5% ± 5.1%, 50 cm: − 4.0% ± 5.0%), suggesting a progressive increment of both actual and mental durations along with the increase in spatial precision.

### Influence of path spatial constraints on both actual and mental movement duration in PwMS

**Figure 2:**
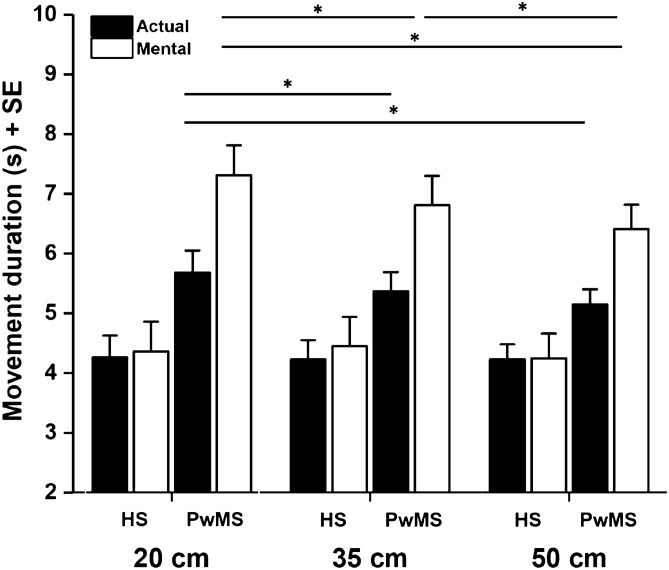
Graphical representation of the group*path width*type of movement interaction. Asterisks indicate significant differences (*p* < 0.05). Mental locomotion was significantly different across all path widths (*p*_*s*_ < 0.05) in PwMS, but not in HS. Considering actually executed locomotion, significant differences were only found between the narrower (20 cm) and the wider paths (35 cm and 50 cm) in PwMS. Vertical lines represent standard error.

Although IP offered a single and informative measure to examine participants’ mental movement ability, it did not clarify whether and to what extent subjects tended to overestimate or underestimate mental movements across paths with different spatial widths. The ANOVA on actual and mental movement duration further qualified the results found in the IP statistical analysis. A significant group*path width*type of movement interaction was found [F (2,56) = 6.801, *p* = 0.040, η2 = 0.108] (Fig. 2): post-hoc analysis showed that actual locomotion across all path widths in PwMS was significantly slower compared to that in HS (PwMS M ± SE: 20 cm: 5.68 ± 0.37 s, 35 cm: 5.37 ± 0.32 s, 50 cm: 5.15 ± 0.25 s; HS M ± SE: 20 cm: 4.26 ± 0.37 s, 35 cm: 4.23 ± 0.32 s, 50 cm: 4.23 ± 0.25 s) (always *p* < 0.05). The same result was found for mental locomotion (PwMS M ± SE: 20 cm: 7.31 ± 0.50 s, 35 cm: 6.81 ± 0.49 s, 50 cm: 6.41 ± 0.41 s; HS M ± SE: 20 cm: 4.36 ± 0.50 s, 35 cm: 4.45 ± 0.49 s, 50 cm: 4.25 ± 0.41 s) (always *p* < 0.005). Moreover, mental locomotion of PwMS was significantly slower with respect to actual locomotion at all path widths (always *p* < 0.001). We did not observe the same result in HS (always *p* > 0.05). In addition, while mental locomotion in PwMS was significantly different across all path widths (always *p* < 0.005), considering actually executed locomotion, significant differences were only found between the narrower (20 cm) and the wider paths (35 cm and 50 cm) (*p* < 0.005 in both cases) (see Fig. 2 for a graphical representation of major results).

### Role of cognitive fatigue on PwMS’ performances

**Figure 3:**
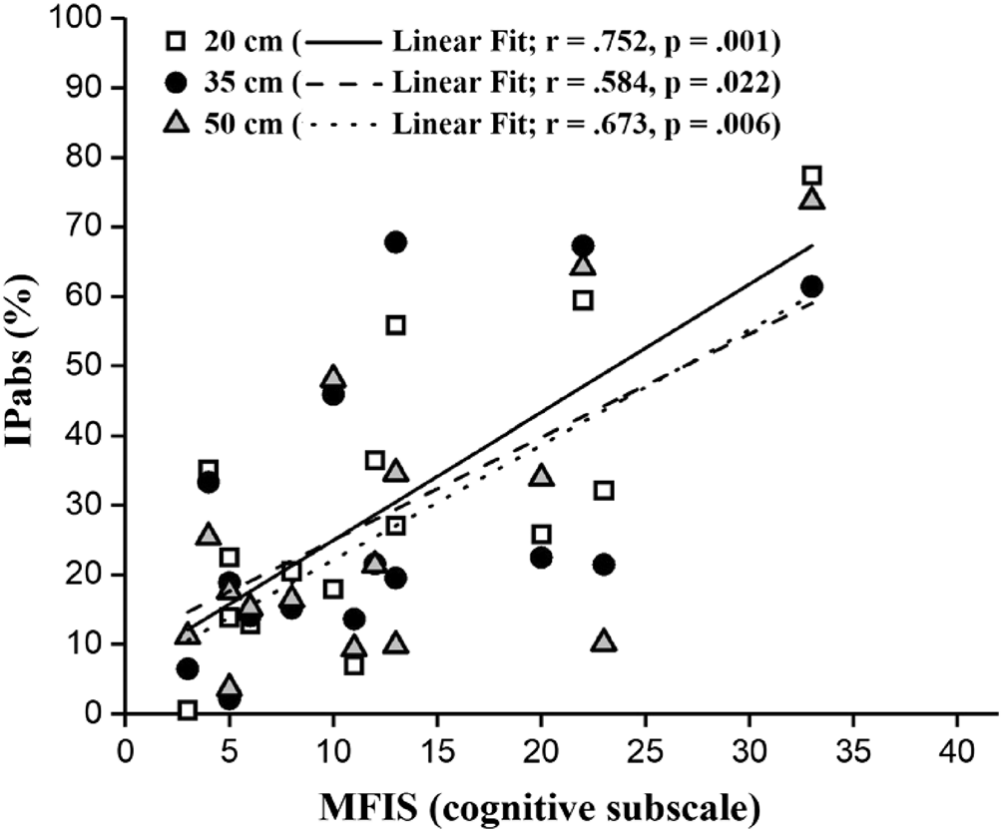
Graphical representation of the correlation between absolute values of IPabs and cognitive subscale of MFIS. Values at 20 (white square), 35 (black circle) and 50 cm (grey triangle) are shown. Trend lines and r and p values are presented for each path width.

In PwMS, the correlation between IP_abs_ and the total score of MFIS was significant at all path widths (20 cm: r = 0.719, *p* = 0.002; 35 cm: r = 0.362, *p* = 0.011; 50 cm: r = 0.707, *p* = 0.003): the higher the reported fatigue perception, the higher the anisochrony. More interestingly, IP_abs_ significantly correlated with the MFIS cognitive subscale at all path widths (20 cm: r = 0.752, *p* = 0.001; 35 cm: r = 0.584, *p* = 0.022; 50 cm: r = 0.673, *p* = 0.006), suggesting a role of cognitive fatigue on PwMS’ performances (see Fig. 3). No correlations with physical (20 cm: r = 0.439, *p* = 0.102; 35 cm: r = 0.479, *p* = 0.071; 50 cm: r = 0.512, *p* = 0.051) or psychosocial (20 cm: r = 0.472, *p* = 0.075; 35 cm: r = 0.354, *p* = 0.195; 50 cm: r = 0.408, *p* = 0.131) subscales were found.

## Discussion

MI is a dynamic state during which the representation of an action is internally reproduced within working memory without any overt output^2^. The aim of the present study was to test MI in people with Multiple Sclerosis (PwMS). Although there is clear and consistent evidence that PwMS may have difficulties in the mental simulation of actions, previous results are far from being conclusive. Thus, a critical advance of these studies was necessary. Here, we investigate if PwMS show anisochrony in a task involving lower limbs and if the dependency on spatial constraints was present. In addition, due to the major role of fatigue in the life of PwMS, the relationship between cognitive fatigue and MI performances of patients was investigated. To this aim, fifteen PwMS with mild disability and 15 age-matched healthy subjects (HS) were required to either actually walk or imagine themselves walking on three paths of the same length, but different widths. When asked to mentally simulate a walking movement along a constrained pathway, PwMS tended to overestimate mental movement duration with respect to actual movement duration, thus confirming the presence of anisochrony. Moreover, the investigation of movement durations revealed that a progressive increment of both actual and mental durations occurred with the augmentation of spatial precision; this increment was more consistent for the mental task at the narrowest paths. Interestingly, in line with previous evidence, cognitive fatigue was found to play a role in the MI of PwMS.

The analysis of actual movement durations across different path widths showed that PwMS were slower than HS. Although no statistical differences were found between groups in T25-FW, this result was expected because MS typically influences postural control and body equilibrium as of the first stages of the disease; thus, ambulation was more challenging for PwMS with respect to HS, especially when walking on a path with spatial constraints was required. This effect is also present in mental locomotion; in fact, the mental simulation of movements reflects the updating of motor representation in line with disease evolution. Thus, the slower the actual movements, the slower mental simulation. Interestingly, PwMS and HS reported to spent almost the same time in walking or in other physical activities, as indicated by IPAQ scores. This further corroborated our results suggesting that PwMS could not have less experience of walking to refer when performing the MI tasks and, thus, anisochrony could not be attributed to differences in physical practice or, conversely, sedentary behaviours.

Furthermore, the temporal requirement of the task (i.e. at a self-paced speed) may explain why here we found that the mental actions were slower than those actually executed in the MS group, similarly to the study using the task of squeezing a ball^17^ and differently from that requiring pointing movements^15^. In the latter, PwMS imagined faster than actual performances, probably as a consequence of two concurrent factors. One is that the difficulty of the task may have led to slowness in the actual movement. The other is they could adopt a simulation strategy so to execute the task as accurately and fast as possible, as they were asked to. This would have prevented them from full integrating the spatial constraints (i.e., path width) during their mental processes and may have resulted in a sped up mental representation^63^. In the present study, the lack of temporal constraints may have induced PwMS to dedicate a longer time to accurately simulating the full-body movements and simultaneously updating the environmental spatial information^5^. Moreover, the cognitive demand of the task could be further burdened by the need to verbally indicate when the shoulders mentally crossed the finish line in order to stop the temporal acquisition. Although it is a simple request, for PwMS it could represent a sort of dual-task condition^64^ with consequent slower mental performances^65^. It seems that imagining themselves walking while considering path constraints (additional cognitive task), that require dividing attentional capacities is more difficult than a single task^66^. However, as indicated by DTQ score, PwMS in our study did not show any difficulties in managing dual-task activities. Thus, anisochrony in MS group could not be explained in terms of DT cost. Also, a factor that may contribute to MI performance is the presence of cognitive impairment^67^, one of the most disturbing and debilitating disorder in MS, present in 43–70% of adults with MS and documented in all MS subtypes^68,69^. Information processing speed, working memory, attention and executive functions are the major cognitive domains affected^70,71^. Therefore, the representation of a mentally reproduced action requires a preserved cognitive functioning^14^, particularly in terms of speed information processing, which involves the ability to both maintain and manipulate information for a certain period. However, in our study, PwMS performed similarly to HS in SDMT, known to be the best predictor of MS cognitive impairment^53^. However, to date, a neuropsychological assessment covering attention, memory, visuo-spatial and executive functions may be adequate to both detect subtle cognitive deficits and to disentangle their possible role in MI.

Beside spatial constraints, fatigue also seems to influence mental performances as demonstrated by a strong correlation between fatigue perception as measured by MFIS and MI ability. Fatigue is likely to affect somato-sensory perception and involve perturbation of the body schema^72,73^, strongly enhancing perception of effort and limiting the endurance of sustained physical and mental activities^74^. MS related fatigue had such a negative impact on the quality of life, daily activities, psychological well-being and relationships with friends. Furthermore, although cognitive fatigue could be related to other different cognitive states as drowsiness, loss of attention, decreased arousal, lower focus level^75^, our results are in line with other studies that confirmed a significant relationship between cognitive fatigue and MI performance^48,76,77^. A fatigued body after either continuous or intermittent exercises may affect MI ability, since it reproduces both forward and inverse prediction models, that are crucial to generate temporally accurate and vivid motor representations. In line with^77^, anisochrony in walking could thus originate from the central integration of proprioceptive afferents under a fatigued state that may impact, in turn, motor predictions of movements involving lower limbs.

At neural level, in MS related fatigue an abnormal recruitment of the primary motor cortex (PMC) and supplementary motor cortex (SMC), two areas involved in movement control, has been recently shown. In addition, microstructural damage in several fronto-connected associative white matter (WM) tracts (i.e., corona radiata, cingulum, anterior thalamic radiation, corpus callosum, forceps minor, superior longitudinal fasciculus, inferior fronto-occipital fasciculus, thalamus, cerebral peduncle and basal ganglia)^47^ has also been highlighted, thus supporting the hypothesis that fatigue is subtended by both a motor and non-motor network dysfunction^46^. Furthermore, the anterior cingulate cortex (ACC), which is extensively connected with other cortical areas and associated to alertness and attention, has been found to be altered in PwMS. Thus, its dysfunction in MS might represent the substrate of cognitive fatigue and/or the cognitive component of MS-related counterparts of fatigue^46,78^. Several studies have interpreted the increased cerebral activity as a rearrangement, a “compensation” occurring in PwMS in an attempt to make up for the neural dysfunction and, thus, responsible for the development of fatigue symptoms^46^. As suggested by^49^, it is possible that while compensation may indeed be occurring, that factor itself might lead to cognitive fatigue. Equally, cognitive fatigue might require the recruitment of additional brain areas to compensate for the extra ‘effort’ required for a continued performance. Although this study lacked neuroimaging data of actual and mental tasks, preventing us from drawing conclusions about the neural mechanism between compensation and cognitive fatigue, we can speculate a pivotal role of cognitive fatigue that might influence the way in which PwMS experienced the mental task, probably affecting its internal representation.

Moreover, many findings have frequently pointed out the similarities between the profile of deficits in MS and that observed in healthy elderly people^79–81^. In the study by Personnier et al.^5^, in which the temporal features of mental locomotion in normal aging were investigated, the authors found that elderly people increased anisochrony when the spatial precision required to perform the task increased. Our study is in line with findings from Personnier et al.^5^ confirming a tendency to anisochrony in PwMS, further corroborated by the investigation of movement durations. We found that a progressive increment of both actual and mental durations occurred with the augmentation of spatial precision; this increment was more consistent for the mental task at the narrowest paths.

Although current results do not have direct therapeutic implications, they provide some critical hints for MI use in the clinical and rehabilitation context. In fact, to date, a plethora of studies demonstrated that mental practice through MI leads to motor improvements by constituting a potential tool for motor learning and relearning, and for rehabilitation, especially for people with physical disabilities^14,82^. Specifically for PwMS, it seems that spatial constraints and cognitive fatigue need to be correctly weighted according to the individual rehabilitative intervention. Since walking is typically one of the first functions to be affected by MS with a high impact on the quality of life of the patients, a better understanding of how PwMS are able to imagine walking could contribute to improving understanding of their action representation and eventually proposing new rehabilitative tools based on the mental practice of balance and locomotion^14^. Improvements in motor task execution (e.g., increased gait speed) following MI training are believed to be due to the development and refining of the internal representation of the motor task via activation of the movement-related neural network^83^. Furthermore, mental practice with MI offers a unique and attractive opportunity to improve locomotor skills through a safe and self-paced training for people with severe disability that, due to their walking difficulties, can be done only in short bursts, especially in the early phase of rehabilitation. Given the potential benefits of MI in neurological rehabilitation, further studies are needed to explore its contribution to the extent of motor gains in a population with physical disabilities.
